# Mortality from prostate cancer in the years 2007–2021 in North Rhine-Westphalia, Germany

**DOI:** 10.1186/s12894-024-01564-y

**Published:** 2024-08-27

**Authors:** Kevin Claaßen, Madeleine Karpinski, Hiltraud Kajüter, Johannes Hüsing, Lennart Möller, Ina Wellmann, Viktor Grünwald, Boris Hadaschik, Peter Albers, Andreas Stang

**Affiliations:** 1 State Cancer Registry North-Rhine Westphalia, Bochum, Germany; 2 Department of Medical Statistics and Epidemiology, Medical School Hamburg, Hamburg, Germany; 3 Department of Nuclear Medicine, University Hospital Essen, Essen, Germany; 4 Department of Urology, University Hospital Essen, Essen, Germany; 5 Department of Urology, University Hospital Düsseldorf, Düsseldorf, Germany; 6 Institute of Medical Informatics, Biometry and Epidemiology, University Hospital Essen, Essen, Germany

**Keywords:** Prostate cancer, North Rhine-Westphalia, Germany, Cancer registry, Mortality, Person years life lost, Proportional mortality ratio

## Abstract

**Background:**

The crude mortality rate and the lifetime mortality risk from prostate cancer in Germany are above international average. However age-standardised mortality and years of life lost per capita from prostate cancer are declining. This study analyses the mortality-related measures for the federal state of North Rhine-Westphalia (NRW) in Germany.

**Methods:**

Based on the cause of death statistics and data from the NRW State Cancer Registry on 45,300 deaths in the years 2007–2021, mortality rates, the lifetime mortality risk from prostate cancer, median age at death and years of life lost are presented. Additionally, the 15 most frequent causes of death of 95,013 patients diagnosed with prostate cancer are reported.

**Results:**

With a stable lifetime mortality risk from prostate cancer, age-standardised mortality and years of life lost per capita are decreasing while crude mortality and median age at death are increasing in NRW. Less than half of the patients die from their prostate cancer. Cancers of the urinary bladder and other urinary organs also occur more frequently as a cause of death than it would be expected based on the age-specific risk in the total population.

**Conclusions:**

More people in North Rhine-Westphalia are dying of prostate cancer over time due to demographic ageing alone. At the same time, the age-specific mortality risk has not increased and when patients die of prostate cancer, it is at an increasingly older age. However, there is a statistical association with deaths from cancers of the lower urinary tract in patients diagnosed with prostate cancer, which demands further evaluation.

## Background

In 2020, prostate cancer was responsible for a roughly estimated 375,000 deaths in the 185 countries of the Global Burden of Cancer (GLOBOCAN) database. This corresponded to four per cent of all cancer-related deaths, making prostate cancer the eighth leading cause of cancer-specific death. This translates to an age-standardised mortality rate of eight deaths per 100,000 person-years (world standard population). The estimated cumulative risk of dying from prostate cancer between birth and 74 years of age was less than one per cent, although competing risks were not taken into account and there were substantial differences between countries [1].

More than half of the deaths in the 185 countries occurred over the age of 65 [2]. Around 109,000 deaths, or 29 per cent, occurred in Europe [3]. In the same study, it is estimated that by 2040, the absolute mortality will increase by 92 per cent to around 720,000 deaths per year. For Europe, an increase in absolute mortality of 53 per cent to 166,000 deaths per year is forecasted. On the other hand, the age-standardised mortality rate (world standard population) has begun to fall around the mid-1990s in the countries of Western Europe (with the observation period of the study cited here ending in 2014) [4].

In Germany, 15,040 men died from prostate cancer in 2019. This corresponded to 12 per cent of all male cancer deaths (excluding ICD-10 code C44), making it the second most common cause of cancer-specific death. The age-standardised mortality rate based on the old European standard population was 19 deaths per 100,000 person-years in Germany. Due to the use of different standard populations, this rate cannot be directly compared to the aforementioned age-standardised mortality rate of eight deaths per 100,000 person years in the GLOBOCAN-dataset. The age-standardised mortality rate in Germany declined between 1999 and 2006. Since then, a relatively constant course has been observed. The median age at death in 2019 was 81 years. Taking into account competing causes of death, the lifetime risk of dying from prostate cancer in Germany was three per cent. The risk of developing the disease was 14 per cent [5].

At the moment, there is no organised screening programme for prostate cancer in Germany. Instead, opportunistic screening with an annual digital rectal examination for men aged 45 and over is recommended and payed for by the health insurance companies. The PSA test is a self-pay service that is also recommended to this population after detailed explanation of the pros and cons of PSA testing [6].

The mortality-related burden of a disease can be measured by the loss of potential life years. For patients who died of prostate cancer in the US in 2015, this resulted in 289,000 potential years of life lost in total and 10 years per deceased patient [7]. Despite a comparatively high incidence, prostate cancer caused only two per cent of the total years of life lost to cancer [8]. A more recent study from Oxford (UK) found an age-standardised 485 years of life lost per 100,000 person-years in the US, 442 (France) to 628 (Netherlands) years of life lost in Western Europe and 585 in Germany [9]. A downward trend has been observed in both the US and Western Europe since 2000. The Robert Koch Institute's BURDEN 2020 project calculated that a total of 3.3 million years of life were lost to cancer in Germany in 2017. Around 200,000 of these, or six per cent, were attributable to prostate cancer as the primary cause of death [10].

Prostate cancer therefore is a disease with a lower mortality rate as compared to other entities, which particularly affects ageing societies. Against this background, this article addresses the following questions in relation to the population of North Rhine-Westphalia (NRW), Germany: How many people die of prostate cancer each year and at what age? How likely is it to die of prostate cancer in the course of a lifetime and how many potential years of life are lost as a result? What causes the death of patients with prostate cancer and how frequently do these causes of death occur in relation to the general population?

## Methods

For this study, data from the NRW State Cancer Registry was used for the years 2007 to 2021. In Germany, the diagnosis, treatment and progression of cancer is subject to statutory reporting requirements by medical service providers since 2005. The database of the NRW state cancer registry represents a population of circa 18 million residents. Its completeness in relation to incident cases is estimated at over 90 per cent across all entities [5]. The NRW State Cancer Registry also functions as a mortality registry that receives death notifications from both the clinicians and the registry offices. The cause of death information on deceased subjects is reported to the cancer registry by the State Office for Information and Technology (IT.NRW).

On this basis, mortality rates, mortality risks, the median age at death and the potential years of life lost due to prostate cancer as well as the most common causes of death and the respective age-standardised and age-specific proportional mortality ratios (PMR) of patients with prostate cancer were calculated. The annual percentage change (APC) over the study period was estimated in order to depict long-term trends over time rather than random variation. This was done by fitting regression lines to the natural logarithm of the annual outcome measures by use of calendar years as the predictor variable. The APC was then calculated based on the regression coefficient (b) as: $APC={(e}^{b}-1)*100$. The 95% confidence intervals of the APCs were derived from a t-distribution [11].

The analyses were based on two different types of registry data. The calculation of mortality rates and risks was based on the official cause of death statistics by (male) sex and 5-year age groups for the mortality years 2007 to 2021. In contrast, the calculation of median age at death, years of life lost, causes of death and proportional mortality ratios was based on data from the state cancer registry to the database status as of June 30, 2023.

All persons considered in the analysis of mortality rate and risk, median age at death and years of life lost died from prostate cancer (ICD-10: C61) in the years 2007 to 2021. They were registered as residents of NRW. For the derivation of the most frequent causes of death and the proportional mortality ratios, the inclusion criterion referred to the diagnosis C61 instead of the cause of death meaning this study cohort consists of patients who were diagnosed with prostate cancer and died subsequently from any cause in the period 2007 to 2021.

A presentation of the study cohorts can be found in Table 1. The number of cases (n) depends on the outcome measure. A difference between the official statistics and the data from the state cancer registry is due to the fact that in rare cases the record linkage between the reports of deaths by the registration offices and the causes of death by IT.NRW was not possible for technical reasons.

**Table 1:** Characteristics of the study cohorts with either death from or diagnosis of prostate cancer. Data from North Rhine-Westphalia, Germany, 2007–2021

Measure	Criteria	Data source	n	Person years
Cumulative mortality risk	Cause of death: C61	Official cause of death statistics	46,354	-
Lifetime mortality risk	Cause of death: C61	Official cause of death statistics	46,354	-
Median age at death	Cause of death: C61	Cancer registry data	45,300	171,898
Years of life lost	Cause of death: C61	Cancer registry data + official cause of death statistics	45,300	171,898
Causes of death	Diagnosis: C61 + Death from any cause	Cancer registry data	95,013	490,140
Proportionate mortality ratios	Diagnosis: C61 + Death from any cause	Cancer registry data + official cause of death statistics	95,013	490,140

The mortality rate by calendar year was calculated on a crude, age-specific and age-standardised basis. The age standardisation was carried out according to the old European standard population [12].

The calculation of the lifetime mortality risk was based on the ‘current probability’ method according to Fay, which takes competing risks into account [13]. In addition, the cumulative mortality risk up to and including 84 years was calculated, which did not correct for competing risks. The ‘DevCan’ programme of the National Institute of Cancer (NIC) was used for the calculation of the lifetime mortality risk [14]. In addition to this software, the statistical analysis was performed using the programme R in version 4.2.1. The estimations of lifetime mortality risk and median age at death were based on non-parametric methods, meaning that symmetrical confidence intervals did not necessarily result.

The calculation of potential years of life lost due to prostate cancer required knowledge of age-specific life expectancies. The latter was calculated individually as the sum of age and remaining life expectancy. This data was taken from the IT.NRW mortality tables [15]. The discontinued remaining life expectancy of over 100-year-olds was equated with that of 100-year-olds. As no mortality tables were available for the year of death 2021, the figures for 2020 were used. The absolute years of life lost for n deceased persons were calculated as follows: $\sum_{i=1}^nLife\;Expectancy-{Age\;at\;Death}_i$.

With regard to the causes of death of patients with prostate cancer, the 15 most common ICD-10 codes were identified at a three-digit level. The age-standardised PMR (for a specific cause of death) was calculated as the ratio between the observed deaths among patients and the expected deaths. The expected deaths are calculated as the sum of the products of the number of people in the study population in the respective age groups and the proportionate mortality in the age-matched reference population of deceased males in NRW. A Poisson distribution was assumed regarding the 95 per cent confidence intervals [16]. An additional age-specific presentation of the PMRs refers to the age at the time of death.

## Results

**Fig. 1:**
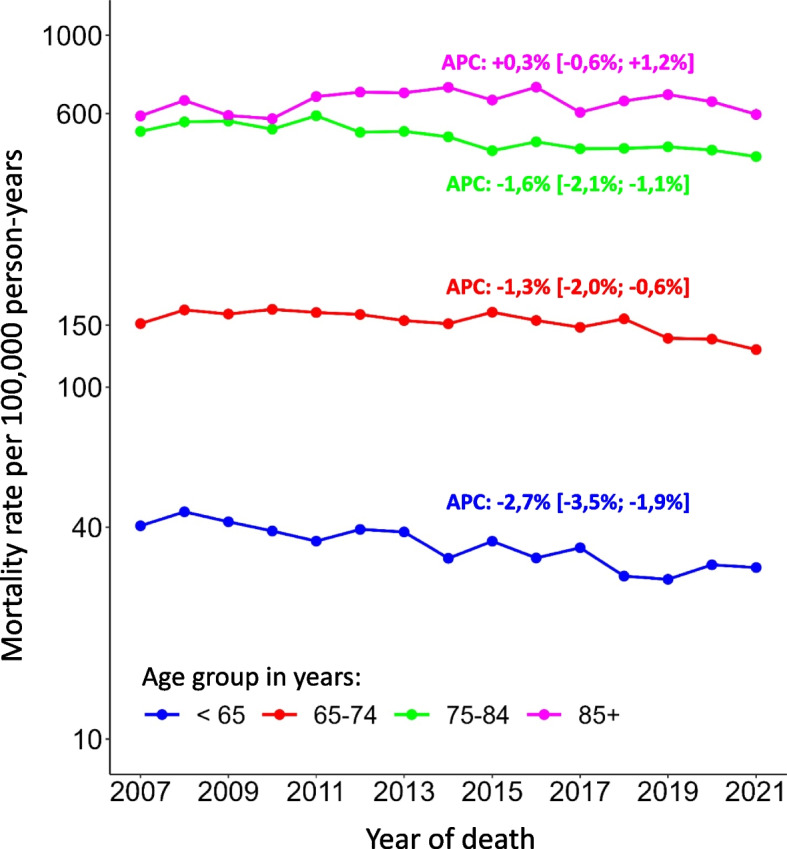
Age-specific prostate cancer mortality rates by year of death. Data from North Rhine-Westphalia, Germany, 2007–2021, *n* = 46,354

Figure 1 shows the annual age-specific mortality rates for prostate cancer per 100,000 men with APCs and 95% confidence intervals. The mortality rate is declining in three of the four age groups. Only the open age group of 85 + years shows a constant trend.

**Fig. 2:**
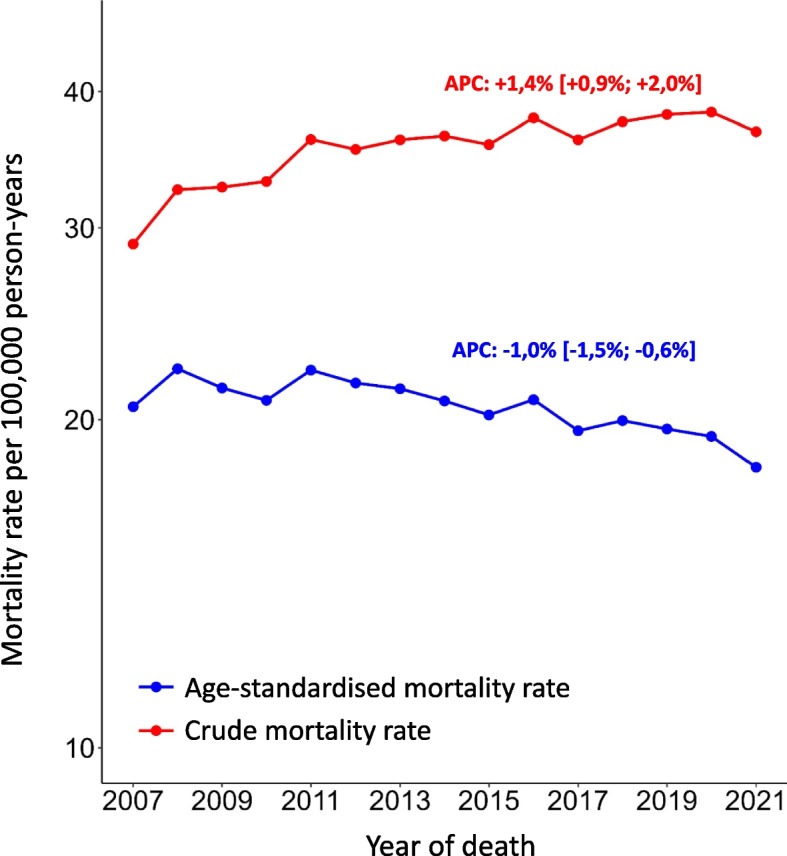
Crude and age-standardised prostate cancer mortality rates by year of death. Data from North Rhine-Westphalia, Germany, 2007–2021, *n* = 46,354

In addition, the crude and age-standardised mortality rates are shown in Fig. 2 with APCs and 95% confidence intervals. While the crude mortality rate rose from 29.0 to 36.8 between 2007 and 2021 (with a maximum in 2020), the age-standardised rate fell from 20.5 to 18.1 over the same period. This means that the age-standardised rate in 2021 corresponds to the minimum of the period under review.

**Fig. 3:**
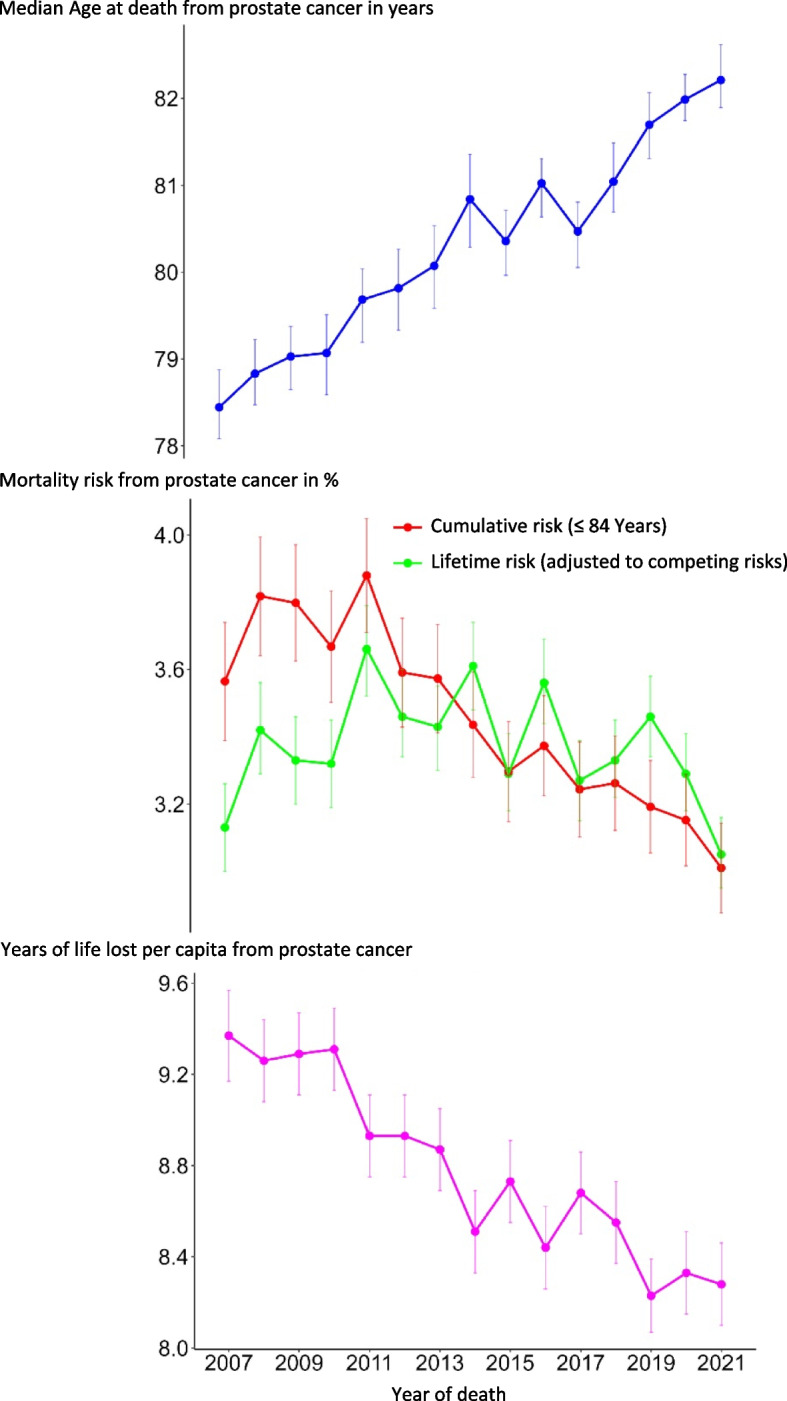
Median age at death, mortality risk and years of life lost to prostate cancer. Data from North Rhine-Westphalia, Germany, 2007–2021; *n* = 45,300 for median age at death and years of life lost per capita, *n* = 46,354 for cumulative and lifetime mortality risk

Figure 3 shows the development over time of the median age at death, the lifetime mortality risk and the years of life lost per capita to prostate cancer with 95% confidence intervals as vertical lines. The median age at death rose from 78.4 to 82.2 years between 2007 and 2021. The APC is + 0.3 per cent (95% CI: [+ 0.3%; + 0.4%]).

The lifetime mortality risk of dying from prostate cancer, taking into account competing causes of death, is about three per cent over time. Although the risk has transiently risen to 3.7 per cent in 2011, the APC is -0.2 per cent [-0.9%; + 0.5%], meaning that no clear time trend is discernible. In contrast, the cumulative mortality risk (up to and including the age of 84 years) decreased by 0.6 percentage points from 3.6 per cent in 2007 to 3.0 per cent in 2021. The resulting APC is -1.6 per cent [-2.0%; -1.2%].

The years of life lost per capita due to prostate cancer have decreased from 9.4 years in 2007 to 8.3 in 2021. The APC is -1.0 per cent [-1.1%; -0.8%]. The development of years of life lost thus mirrors that of the median age at death. Table 2 contains the numerators and denominators of these years of life lost. I. e. the absolute years of life lost by year of death in NRW and the respective number of deceased persons with prostate cancer as the cause of death.

**Table 2:** Years of life lost to prostate cancer and number of deaths by year of death. Data from North Rhine-Westphalia, Germany, 2007–2021, *n* = 45,300

**North Rhine-Westphalia, Germany (*n* = 45,300)**		
**Year of death**	**Years of life lost**	**# Deaths**
2007	23 474	2 506
2008	26 100	2 820
2009	25 968	2 795
2010	26 386	2 833
2011	27 449	3 074
2012	25 956	2 907
2013	26 431	2 980
2014	26 368	3 100
2015	26 804	3 071
2016	27 284	3 233
2017	27 137	3 125
2018	27 680	3 236
2019	27 117	3 295
2020	26 580	3 190
2021	25 969	3 135

Overall, 612 patients with incident prostate cancer died from COVID-19, 249 patients in 2020 and 393 patients in 2021. This corresponds to 2.8 and 4.3 per cent respectively of all patients with incident prostate cancer who died in the according year (CAVE: unlike previously, C61 as inclusion criterion for this analysis refers to the diagnosis and not the cause of death).

Table 3 illustrates the 15 most common causes of death of incident prostate cancer patients who died between 2007 and 2021 with absolute and relative frequencies, PMRs and 95% confidence intervals (CI). Less than half of the 95,013 patients (44.5%) died from prostate cancer. Specifically, the PMR is 12.0, which means that the number of deaths in the study population is 12 times higher than the number that would be expected based on their age structure and the proportionate mortality in the reference population. Further PMRs greater than one result for cancers of the urinary bladder (C67: 1.71) and ‘other and unspecified urinary organs’ (C68 including the urethra, C68.0: 2.31). The remaining 12 of the 15 most common causes of death occur less frequently within the study population than would be expected based on its age structure resulting in PMRs below one.

**Table 3:** Frequency of the 15 most common causes of death for patients with prostate cancer. Data from North Rhine-Westphalia, Germany, 2007–2021, *n* = 95,013

**North Rhine-Westphalia, Germany (*n* = 95,013)**			
**ICD-10-Code**	**Unicausal cause of death**	**% (n)**	**PMR [95% CI]**
C61	Prostate cancer	44,5% (42 256)	12,0 [11,9; 12,2]
I25	Chronic ischemic heart disease	4,4% (4 169)	0,6 [0,5; 0,6]
C34	Malignant neoplasm of the bronchi and lungs	3,7% (3 542)	0,5 [0,5; 0,6]
I21	Acute myocardial infarction	2,6% (2 509)	0,5 [0,47; 0,51]
J44	Other chronic obstructive pulmonary disease	2,3% (2 146)	0,5 [0,49; 0,54]
I50	Heart failure	2,2% (2 059)	0,5 [0,50; 0,54]
R99	Other imprecise or unspecified causes of death	1,8% (1 714)	0,5 [0,5; 0,6]
C67	Malignant neoplasm of the urinary bladder	1,7% (1 590)	1,7 [1,6; 1,8]
C68	Malignant neoplasm of other and unspecified urinary organs	1,5% (1 452)	2,3 [2,2; 2,4]
C25	Malignant neoplasm of the pancreas	1,4% (1 315)	0,8 [0,7; 0,8]
F03	Unspecified dementia	1,2% (1 151)	0,5 [0,48; 0,54]
C18	Malignant neoplasm of the colon	1,2% (1 111)	0,6 [0,55; 0,61]
J18	Pneumonia, pathogen unspecified	1,1% (1 082)	0,5 [0,46; 0,52]
C80	Malignant neoplasm without indication of location	1,1% (1 008)	0,9 [0,8; 0,9]
I63	Stroke	0,9% (813)	0,5 [0,47; 0,54]

Similarly, the age-specific analysis in Table 4 shows PMRs above one only for C61, C67 and C68. For these ICD-10 three-digit codes, there is an increase in all age groups compared to the expected number of cases. In the group of under 65-year-olds, the number of deaths due to cancer of the urinary bladder and other urinary organs is increased by a factor of around five compared to the expected number of cases. The PMRs for the remaining causes of death stay below or equal one in all age groups. With increasing age, the observed deaths increasingly approach the expected number.

**Table 4:** Proportional mortality ratios of the 15 most common causes of death for patients with prostate cancer. Data from North Rhine-Westphalia, Germany, 2007–2021, *n* = 95,013

**North Rhine-Westphalia, Germany (*n* = 95,013)**							
		**PMR [95% CI]**					
**ICD-10-Code**	**Unicausal cause of death**	** < 65 Years**	**65–69 Years**	**70–74 Years**	**75–79 Years**	**80–84 Years**	** > 85 Years**
C61	Prostate cancer	38,9 [37,5; 40,4]	18,3 [17,7; 19,0]	13,0 [12,7; 13,4]	10,6 [10,4; 10,8]	10,1 [9,9; 10,3]	11,6 [11,4; 11,8]
I25	Chronic ischemic heart disease	0,4 [0,3; 0,5]	0,4 [0,4; 0,5]	0,5 [0,4; 0,5]	0,5 [0,5; 0,6]	0,6 [0,56; 0,63]	0,6 [0,55; 0,61]
C34	Malignant neoplasm of the bronchi and lungs	0,4 (0,3; 0,4]	0,5 (0,4; 0,5]	0,5 (0,5; 0,6]	0,6 (0,5; 0,6]	0,6 (0,5; 0,6]	0,6 (0,6; 0,7]
I21	Acute myocardial infarction	0,3 (0,3; 0,4]	0,4 (0,3; 0,5]	0,4 (0,4; 0,5]	0,5 (0,5; 0,6]	0,5 (0,5; 0,6]	0,5 (0,5; 0,6]
J44	Other chronic obstructive pulmonary disease	0,4 (0,3; 0,5]	0,4 (0,4; 0,5]	0,4 (0,4; 0,5]	0,5 (0,5; 0,6]	0,6 (0,5; 0,6]	0,6 (0,5; 0,6]
I50	Heart failure	0,5 (0,3; 0,7]	0,4 (0,3; 0,6]	0,5 (0,4; 0,6]	0,6 (0,5; 0,6]	0,5 (0,5; 0,6]	0,5 (0,46; 0,53]
R99	Other imprecise or unspecified causes of death	0,3 [0,2; 0,4]	0,4 [0,4; 0,5]	0,5 [0,5; 0,6]	0,6 [0,5; 0,6]	0,7 [0,6; 0,7]	0,6 [0,6; 0,7]
C67	Malignant neoplasm of the urinary bladder	4,8 [4,0; 5,6]	2,6 [2,2; 3,1]	2,3 [2,0; 2,6]	1,9 [1,7; 2,0]	1,5 [1,4; 1,7]	1,0 [0,9; 1,1]
C68	Malignant neoplasm of other and unspecified urinary organs	5,4 [4,6; 6,4]	3,0 [2,5; 3,5]	2,5 [2,2; 2,9]	2,4 [2,2; 2,7]	1,9 [1,7; 2,2]	1,6 [1,4; 1,9]
C25	Malignant neoplasm of the pancreas	0,6 [0,4; 0,7]	0,7 [0,6; 0,8]	0,8 [0,7; 0,9]	0,8 [0,7; 0,9]	0,9 [0,8; 1,0]	0,9 [0,8; 1,1]
F03	Unspecified dementia	0,1 [0,0; 0,8]	0,4 [0,2; 0,7]	0,3 [0,2; 0,5]	0,6 [0,5; 0,6]	0,6 [0,5; 0,6]	0,5 [0,5; 0,6]
C18	Malignant neoplasm of the colon	0,3 [0,2; 0,5]	0,5 [0,4; 0,6]	0,5 [0,4; 0,6]	0,6 [0,6; 0,7]	0,7 [0,6; 0,8]	0,6 [0,5; 0,7]
J18	Pneumonia, pathogen unspecified	0,5 [0,3; 0,8]	0,4 [0,3; 0,6]	0,5 [0,4; 0,6]	0,5 [0,4; 0,5]	0,5 [0,5; 0,6]	0,5 [0,4; 0,5]
C80	Malignant neoplasm without indication of location	1,0 [0,8; 1,2]	1,0 [0,8; 1,2]	0,9 [0,8; 1,0]	0,9 [0,8; 1,1]	0,8 [0,7; 0,9]	0,7 [0,6; 0,8]
I63	Stroke	0,4 [0,2; 0,6]	0,5 [0,4; 0,7]	0,5 [0,4; 0,6]	0,5 [0,4; 0,6]	0,6 [0,5; 0,6]	0,5 [0,4; 0,5]

## Discussion

In 2021, only in absolute terms more people died of prostate cancer in NRW than in 2007. As the standardisation shows, this is due to the demographic ageing of the male population. The consequence is also a higher average age within the 85 + age group, which could explain why the age-specific mortality rate has only remained constant in this group and has not fallen. If the crude annual mortality rates are age-standardised, a discrete decline in mortality of about one percent per year on average can be seen. The lowest age-standardised mortality rate is found in the last year of the observation period (2021) with 18.1 deaths due to prostate cancer per 100,000 person-years. This temporal trend runs counter to the course of the crude (non-standardised) mortality rate.

A similar, as yet unpublished paper by Karpinski et al. deals with the incidence of prostate cancer in NRW and for a longer period of time in the NRW administrative district of Muenster (Karpinski MJ, Claassen K, Möller L, Hüsing J, Kajüter H, Fendler WP, et al.: Incidence and survival of patients with prostate cancer in North-Rhine Westphalia, Germany, submitted). The age-standardised incidence rate in Muenster clearly rose from 59.6 to 133.9 cases per 100,000 person years between 1992 and 2008. From then until 2019, relatively balanced upward and downward changes were observed in Muenster and NRW, after official recommendations against PSA screening at an older age have been issued. The increase in the age-standardised incidence rate until 2008 is not reflected in our mortality data. This is in line with the trend described by Bray et al. that a diminishing association between the (age-standardised) incidence and mortality rate has been observed over the mortality years 1993, 2003 and 2010 in Western Europe [4].

A difference between the official cause of death statistics and the deaths from prostate cancer stored in the cancer registry data was mentioned at the beginning. However, because the difference remains roughly the same over the years, the temporal trend estimates are not expected to be biased.

In 2020 and 2021, COVID-19 was another potential cause of death for patients with prostate cancer, accounting for 2.8 per cent and 4.3 per cent of deaths respectively according to the cause of death statistics. In the two years, a total of 19,530 people in NRW died from COVID-19 [17]. This corresponds to 3.4 and 5.6 per cent of all deaths respectively, so that no increased risk for patients with prostate cancer compared to the overall population can be derived from this.

While the median age at death from prostate cancer increases within the course of the study period, the lifetime mortality risk remains constant. The latter is to be understood as a virtual longitudinal section, because the current mortality risks in the respective age groups are used to predict the lifetime risk of a newborn today. This means that future improvements due to medical-technical progress in the field of therapies or the possible introduction of risk-based screening are excluded.

The cumulative mortality risk from prostate cancer decreases over time because the mortality risk within most of the respective age groups is declining. The availability of more precise and effective diagnostic and therapeutic options for higher-risk prostate cancers possibly contributes to this dynamic. However, this was not analysed as part of the present study. At the same time, it becomes more likely to reach the older age groups (in which there is a higher risk), because competing causes of death also occur less frequently. This results in the aforementioned constant lifetime mortality risk, the calculation of which takes into account the development of these competing causes of death.

The increasing median age at death indicates that there has been a shift in deaths from prostate cancer towards older age groups. Although, it is virtually just as probable to die from prostate cancer in 2021 as it has been in 2007, which can be seen from the missing dynamic in the lifetime mortality risk. The additional consideration of the decline in years of life lost suggests that the life expectancy of persons who ultimately die from prostate cancer has increased more than the life expectancy in the general population. The finding by Karpinski et al. that the relative 5-year survival probability in the NRW administrative district of Muenster improved notably between the 2000–2004 and 2005–2009 diagnosis periods is consistent with this (Karpinski MJ, Claassen K, Möller L, Hüsing J, Kajüter H, Fendler WP, et al.: Incidence and survival of patients with prostate cancer in North-Rhine Westphalia, Germany, submitted).

For more in-depth future research approaches, it is advisable to consider not only quantitative measures like survival and years of life lost due to prostate cancer. Also of interest are the years from the onset of the disease, which are spent with a reduced quality of life as a result.

Slightly less than half of the patients with prostate cancer (45%) die from their condition and not from other causes. This could be due to the fact that opportunistic PSA screening also detects low-grade prostate cancers, with some of them falling into the category of overdiagnosis.

The PMRs show that twelve of the 15 most common causes of death in patients diagnosed with prostate cancer in NRW occur less frequently than in the reference population deceased in the same age. This is primarily an expression of the competition among the different causes of death (if more people die from prostate cancer, less people can die from other causes).

Not only prostate cancer, but also cancers of the urinary bladder and other urinary organs occur more frequently as a cause of death than expected. With a relatively increased risk of 70 and 130 per cent respectively, they are the eighth and ninth most common causes of death in patients with prostate cancer. With increasing age, the number of observed deaths approaches the expected value of the reference population, which indicates that there is a statistical association between the incidence of prostate cancer and urinary tract cancer as the later cause of death, particularly in the younger age groups. However, the design of the present observational study does not allow any causal conclusions to be drawn.

In their review of clinical studies, Kinoshita et al. refer to the incidence and describe an association between the synchronous and metachronous occurrence of prostate and bladder cancer. Prostate cancer after bladder cancer was described more frequently than vice versa. However, the analysis of US, Swiss, Danish, Finnish and Australian cancer registry data, which is also described in the review, does not provide a uniform picture [18].

On the one hand, it is conceivable that the diagnosis and treatment of one malignancy increases the probability of a second being detected in the sense of a detection bias. For example, incidental prostate cancer is regularly diagnosed during radical cystoprostatectomy for bladder cancer [19]. On the other hand, there is strong evidence from epidemiological studies of a moderately increased risk of secondary primary tumours of the bladder after percutaneous radiotherapy of the prostate. The increased risk of bladder cancer is evident both compared to patients who have undergone radical prostatectomy (without radiotherapy) as well as compared to the general population [20–22].

While other authors also note an increase in risk, they point out that the absolute incidence risk for secondary malignancies is too low to justify disregarding radiotherapy [23, 24]. There is also an increased risk of secondary malignancies after androgen deprivation therapy [25]. However, as there is a need for further research, a possible late toxicity of percutaneous radiotherapy of the prostate should be taken into account, particularly in patients with a longer remaining life expectancy, and should at least lead to a close monitoring of the urinary bladder.

The association between prostate cancer and death from cancer of other urinary organs (C68) is also striking. With regard to malignancies of the prostatic urethra, a misclassification in the documentation of the post-mortem examination could possibly cause a bias. While the prostatic part of the urethra is coded as C61 according to ICD-10, this topography corresponds to the code C68 in ICD-O-3 (International Classification of Diseases for Oncology). Nevertheless, most cancers of other urinary organs in NRW are coded as non-specific (C68.9).

## Conclusions

To summarise, more people in North Rhine-Westphalia are dying of prostate cancer over time due to demographic ageing alone. At the same time, the age-specific mortality risk has not increased and when patients die of prostate cancer, it is at an increasingly older age. However, there is a statistical association with deaths from cancers of the lower urinary tract in patients diagnosed with prostate cancer, which demands further evaluation.

## Data Availability

The datasets analysed during the current study are not publicly available unless they are requested according to section 23 of the NRW State Cancer Register Act.
